# SNX12 Role in Endosome Membrane Transport

**DOI:** 10.1371/journal.pone.0038949

**Published:** 2012-06-15

**Authors:** Véronique Pons, Cansel Ustunel, Corinne Rolland, Eleonora Torti, Robert G. Parton, Jean Gruenberg

**Affiliations:** 1 Department of Biochemistry, University of Geneva, Geneva, Switzerland; 2 INSERM, UMR 1048, Institut Maladies Métaboliques et Cardiovasculaires, Université de Toulouse III, Toulouse, France; 3 INSERM, U1043, UPS, Centre de Physiopathologie de Toulouse Purpan (CPTP), Toulouse, France; 4 Institute for Molecular Bioscience and Center for Microscopy and Microanalysis, University of Queensland, Brisbane, Australia; Institut Curie, France

## Abstract

In this paper, we investigated the role of sorting nexin 12 (SNX12) in the endocytic pathway. SNX12 is a member of the PX domain-containing sorting nexin family and shares high homology with SNX3, which plays a central role in the formation of intralumenal vesicles within multivesicular endosomes. We found that SNX12 is expressed at very low levels compared to SNX3. SNX12 is primarily associated with early endosomes and this endosomal localization depends on the binding to 3-phosphoinositides. We find that overexpression of SNX12 prevents the detachment (or maturation) of multivesicular endosomes from early endosomes. This in turn inhibits the degradative pathway from early to late endosomes/lysosomes, much like SNX3 overexpression, without affecting endocytosis, recycling and retrograde transport. In addition, while previous studies showed that Hrs knockdown prevents EGF receptor sorting into multivesicular endosomes, we find that overexpression of SNX12 restores the sorting process in an Hrs knockdown background. Altogether, our data show that despite lower expression level, SNX12 shares redundant functions with SNX3 in the biogenesis of multivesicular endosomes.

## Introduction

Endocytosis is a process by which solutes, growth factors as well as plasma membrane proteins and lipids are internalized into the cell, through clathrin-coated vesicles and clathrin-independent pathways [1]. After reaching early endosomes, molecules are efficiently sorted into different routes. Some molecules including the transferrin receptor are recycled back to the plasma membrane to be reused, while others are routed towards the Golgi complex. Ubiquitinated signaling receptors, including the EGF receptor (EGFR), are targeted to the degradative pathway. In this process, EGFR is sorted into the intralumenal vesicles that form in multivesicular regions of early endosomes via Hrs and ESCRT complexes [2]. The multivesicular regions then detach – or mature – from early endosomes and become multivesicular endosomes, referred to as endosomal carrier vesicles or multivesicular bodies (ECV/MVBs) [3], [4], which function as transport intermediates towards late endosomes. Eventually, intralumenal vesicles and their protein cargo are delivered to lysosomes where degradation occurs.

Sorting nexins (SNX) belong to a family of proteins, which all share a PX domain that binds 3-phosphorylated inositides, thus allowing selective membrane association primarily to endosomes [5], [6] [7]. Beyond this single common characteristic, SNX proteins are involved in various membrane-related processes in the endocytic pathway, and they may contain one or more additional structural motifs with very diverse functions, including protein-protein or protein-lipid interactions, or enzymatic activities [7]. In particular, some SNX family members contain, in addition to the PX domain, a BAR domain, which interacts with membrane lipids and functions as a curvature sensor [6], [8]. This group includes SNX1, SNX2, SNX5 and SNX6, which are components of the retromer complex involved in endosome-to-Golgi transport [9], and SNX4, which regulates transferrin receptor sorting [10]. The PX-BAR group also includes SNX9, which contains an SH3 domain in addition to the BAR domain and which couples actin dynamics and endocytic membrane remodeling [11], [12], SNX18, an SNX9 paralog, involved in AP1-membrane tubulation [13], and SNX33, which plays a role in actin polymerization via interactions with WASP [14]. In addition, SNX13 and SNX25, which share two putative trans-membrane domains and a RGS (regulator of G-protein signaling) domain [7], play a role in mouse development and endocytosis [15] and TGFbeta signaling [16], respectively. Moreover, SNX15, which includes a MIT (microtubule interacting and trafficking molecule) domain is involved in endosome morphology and trafficking [17], while SNX16, which also contains a structural domain with unknown functions, plays a role in EGF receptor trafficking [18] and dynamics of late endosome tubulo-cisternal membranes [19]. Finally, both SNX17 and SNX27 share a C-terminal FERM domain, with SNX17 being involved in LRP recycling [20], and SNX27 in PDZ-directed sorting from endosomes to the plasma membrane [21].

SNX3 belongs to the sub-group of the SNX proteins that contain only a PX domain and no other structural motifs [7]. In recent studies, SNX3 was proposed to play different, presumably complementary, functions in the endosomal system. In yeast, the SNX3 homolog Grd19p is involved in the selective retrieval of some membrane proteins from the pre-vacuolar compartment, presumably late endosomes, to the TGN [22], [23]. Similarly, SNX3 was proposed to be part of an alternative retromer pathway involved in the very specific retrograde transport of Wnt-binding protein Wntless but not other retromer cargo [24], consistent with observations that endosome-to-Golgi transport of Shiga toxin B-subunit does not seem to depend on SNX3 [25], but SNX1 [26]. Evidence also showed that SNX3 is involved in the maturation of the Salmonella-containing vacuole in infected cells [27]. Finally, we had previously reported that SNX3 plays a role in ECV/MVB biogenesis, and controls the formation of intralumenal vesicles that contain EGFR [25]. SNX12 shares the highest homology among the SNX family with SNX3, but nothing is known about its function. In this paper, we investigated the role of SNX12, and report that protein acts as a functional homolog of SNX3 in ECV/MVB biogenesis.

## Results and Discussion

### SNX12 Localizes to Early Endosomes

**Figure 1:**
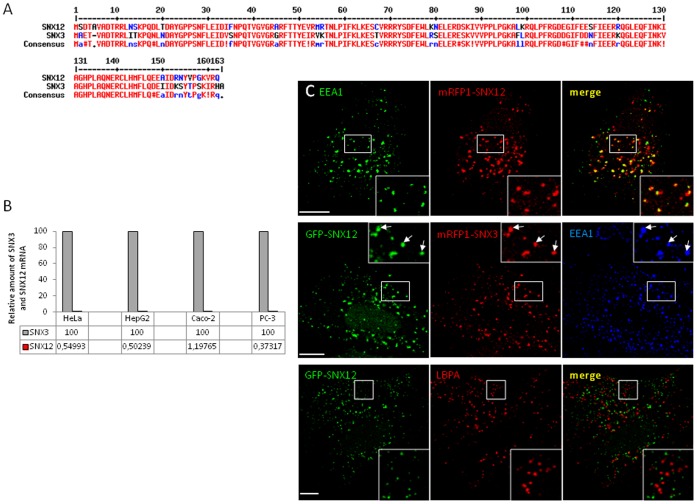
SNX12 is less expressed than SNX3 but is also localized on early endosomes. (**A**) Alignment of amino acid sequences of Homo sapiens SNX3 and SNX12. (**B**) RNA was extracted from different cell lines as indicated and relative amounts of endogenous SNX3 and SNX12 mRNA were quantified by RT-PCR. Values are indicated in the table below the graph since they are very low for SNX12 mRNA. (**C**) HeLa cells expressing GFP-SNX12 or co-expressing GFP-SNX12 and mRFP1-SNX3 were processed for immunofluorescence using the indicated antibodies. Scale bar indicates 10 µm.

SNX12 (NCBI Reference Sequence: NM_013346.2) is a member of the sorting nexin protein family, which apparently contains a PX domain as sole structural domain. The protein consists of 162 amino acids with a predicted molecular weight of ∼20 kDa. Alignment reveals that SNX12 and SNX3, which is also a PX-only protein, share the highest identity amongst SNX family members, with 70.8% DNA identity and 79.5% protein identity (Figure 1A). Since we previously studied the role of SNX3 in protein sorting and membrane transport, we investigated whether SNX12 could also share the same functions as SNX3. To this end, we first quantified by quantitative PCR the relative amount of SNX12 and SNX3 transcripts in different cell lines such as HeLa, HepG2, Caco-2 and PC-3 cells. We found that in these cell lines, SNX12 is expressed at very low levels when compared to SNX3 (≈100–200 fold less) (Figure 1B). This is in good agreement with an exhaustive analysis of the transcription profiles of SNX3 and SNX12 in tissues and cell lines carried out with Genevestigator (https://www.genevestigator.com/gv/index.jsp), which combines thousands of microarray experiments. Interestingly, SNX3 and SNX12 genes are highly conserved in vertebrates. However, only one common ancestor gene is found in insects (D. melanogaster) and yeast (S. cerevisiae), suggesting a gene duplication during evolution. In human, it is important to note that SNX3 gene is located on chromosome 6 (position 6q21) whereas SNX12 gene is located on chromosome X (location Xq13.1). It is thus possible that the expression of the SNX12 gene on sex chromosome is more tightly regulated than that of SNX3 on an autosomal chromosome.

Because of its low abundance and similarity to SNX3, it is not easy to analyze the distribution of endogenous SNX12. We thus tagged SNX12 with GFP and expressed the construct in HeLa cells. Like most PtdIns3P-binding proteins that have been studied, GFP-SNX12 was primarily associated with early endosomes and colocalized with both EEA1 and SNX3, but not with the late endosomal marker LBPA (Figure 1C). Moreover, and as previously described for SNX3 and other PtdIns3P-binding proteins, the endosomal localization of SNX12 was dependent on the binding to 3-phosphoinositides since treatment with wortmannin, a pan-inhibitor of PI 3-Kinases, induced the release of SNX12 in the cytoplasm (Figure S2A). Release was not complete presumably because the proteins remained in part associated to membranes via protein-protein interactions. To analyse more precisely the role of the PX domain in membrane association, we mutated the highly conserved arginine 71 of the PX domain of SNX12 to alanine. The protein was incubated with PtdIns3P-containing liposomes, and then the mixture was loaded at the bottom of a step sucrose gradient and liposomes were retrieved by floatation. We observed that the SNX12^R71A^ mutant had lost the capacity to bind PtdIns3P-containing liposomes, when compared to wild type SNX12 (Figure S2B-C). Consistently, the PtdIns3P binding-defective mutant SNX12^R71A^, was no longer membrane-associated (Figure S2A), much like SNX3^R70A^ mutant [25], consistent with the notion that membrane targeting requires PtdIns3P-binding.

A recent study showed that SNX3 interacts with the retromer and is involved in the highly selective retrograde transport of the Wnt sorting receptor Wntless [24]. We thus investigated whether SNX12 may also be linked to the retromer components. We observed by immunofluorescence that mRFP1-SNX12 co-localizes with the retromer subunits Vps35 and Vps26 on endosomes (Figure S3A). We then investigated whether SNX12 interacts with the retromer using GST pull down assays. After incubating cell lysates with recombinant SNX1, SNX3 or SNX12, we found that, as expected, SNX1 and SNX3 binds to Vps35. Interestingly, SNX12 is also associated with Vps35, demonstrating that SNX12 shares with SNX3 the capacity to interact with retromer components (Figure S3B-C).

### Excess SNX12 Inhibits Transport Beyond Early Endosomes

**Figure 2:**
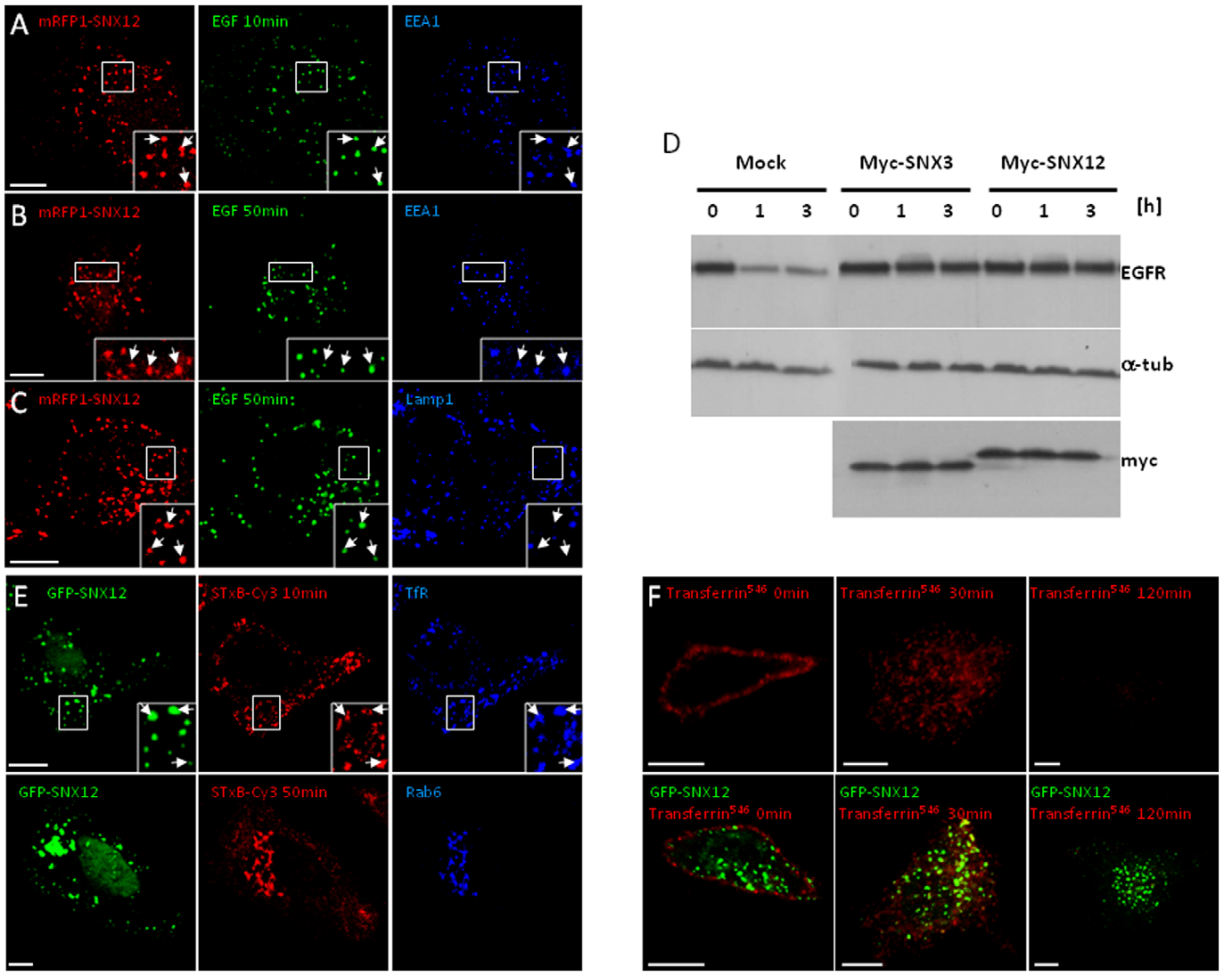
SNX12 overexpression inhibits EGFR transport and degradation without affecting retrograde and recycling transport routes. (**A-C**) After cell surface binding, EGF-biotin coupled to streptavidin^AlexaFluor488^ was endocytosed for 10 min (**A**) or 50 min (**B-C**) at 37°C in HeLa cells expressing mRFP1-SNX12. Cells were labeled with anti-EEA1 (**A**) or Lamp1 (**B-C**) antibodies and analyzed by triple channel fluorescence. (**D**) HeLa cells expressing myc-SNX12 or myc-SNX3 or mock-treated were incubated with EGF for the indicated time periods. Cell lysates (100 µg) were analyzed by SDS gel electrophoresis and western blotting with antibodies against EGFR, α-tubulin (a-tub) or myc**.** (**E**) After cell surface binding, Shiga toxin B-subunit conjugated to Cy3 was internalized for 10 min or 50 min at 37°C into HeLa cells expressing GFP-SNX12. Cells were labeled with anti-transferrin receptor (TfR) or Rab6 antibodies and analyzed by triple channel fluorescence. (**F**) After cell surface binding (0 min), transferrin conjugated to AlexaFluor546 (transferrin^546^) was internalized for 30 min or 120 min at 37°C into control cells (upper panels) or cells expressing GFP-SNX12 (lower panels). Cells were then analyzed by fluorescence. (**A-C and E-F**) Scale bar indicates 10 µm.

Previously we had observed that excess SNX3 inhibited membrane transport beyond early endosomes. We then tested whether SNX12 overexpression caused a similar inhibition. When overexpressed, SNX12 did not affect EGF internalization since endocytosed EGF^AlexaFluor488^ colocalized with EEA1-positive early endosomes after 10 min pulse (Figure 2A) much like in control cells (Figure S1A). However, and in contrast to control cells (Figure S1B-C), EGF was not transported to Lamp1-positive late endosomes (Figure 2C) but still remained in early endosomes (Figure 2B) after 50 min in cells expressing SNX12. Consistent with these observations, overexpressed Myc-SNX12 inhibited EGFR degradation in the presence of EGF to the same extent as myc-SNX3, at comparable levels of expression (Figure 2D). By contrast, the PtdIns3P binding-defective mutant SNX12^R71A^ did not affect EGFR degradation (Figure S2D). This demonstrates that SNX12^R71A^ mutant, which is not able to bind PtdIns3P, having lost the capacity to localize to endosomes, cannot interfere EGFR transport and degradation.

Having observed that overexpressed SNX12 inhibited transport towards late endosomes and lysosomes, we investigated whether all export routes from early endosomes were also affected. Shiga toxin B-subunit navigates retrogradely, from the plasma membrane via early/recycling endosomes, to the Golgi apparatus and eventually to the endoplasmic reticulum. Shiga toxin B-subunit reached transferrin receptor-positive early endosomes after 10 min incubation at 37°C and was then exported to the Golgi complex containing Rab6 after 50 min in cells expressing SNX12 (Figure 2E) as well as in control cells (Figure S1D). This demonstrates that Shiga toxin B-subunit retrograde transport from early endosomes to the Golgi apparatus is not affected by excess SNX12.

Similarly, binding of fluorescent transferrin to its receptor on the cell surface was much the same as in cells overexpressing GFP-SNX12 and control cells (Figure 2F), indicating that receptor cycling was most likely not affected by overexpression. Upon incubation for 30 min at 37°C, transferrin, like EGF (Figure 2A), was efficiently internalized in cells overexpressing GFP-SNX12 as in control cells (Figure 2F), further demonstrating that endocytosis was not affected. When cells were incubated at 37°C for longer time periods, no transferrin could be detected intracellularly whether SNX12 was overexpressed or not (Figure 2F), demonstrating that transferrin receptor recycling was not affected by excess SNX12.

It thus appears that SNX12 overexpression specifically blocks the degradative pathway from early to late endosomes/lysosomes, without affecting endocytosis, recycling from early endosomes to the plasma membrane and retrograde transport from early endosomes to the Golgi apparatus.

### Infection with Vesicular Stomatitis Virus

**Figure 3:**
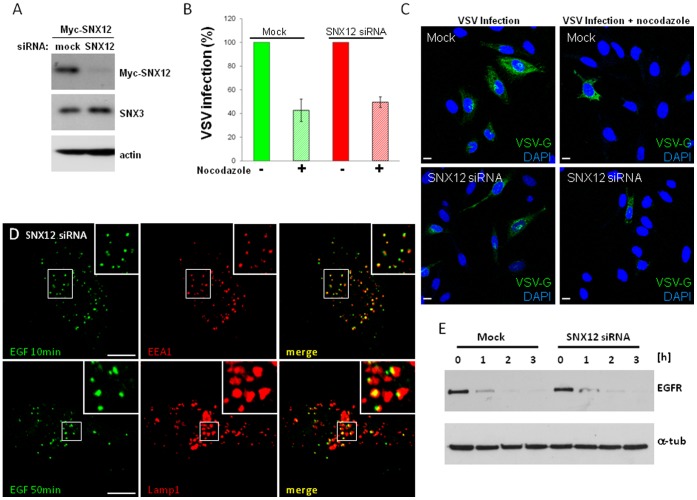
SNX12 silencing has no effect on VSV infection and EGFR transport and degradation. (**A**) HeLa cells treated with siRNAs against SNX12 or mock-treated were lysed. Lysates were analyzed by SDS gel electrophoresis and western blotting using with antibodies against myc, SNX3 or actin. (**B-C**) HeLa cells were treated with SNX12 siRNAs or mock-treated, and then microtubules were depolymerized or not with 10 µM nocodazole for 2 h. VSV (1 MOI) was bound at 4°C at the cell surface and cells were then incubated for 3 h at 37°C to allow VSV infection to proceed. Cells were analyzed by immunofluorescence with antibodies against VSV-G protein. Scale bar indicates 10 µm. Experiments were quantified (**B**) and representative pictures were shown in (**C**). Under these conditions ≈50% of the control cells were infected so that changes in infection rate can be best monitored, and values are normalized to the controls. Each condition is the mean of at least three independent experiments; standard errors are indicated. (**D**) After cell surface binding, EGF-biotin coupled to streptavidin^AlexaFluor488^ was endocytosed for 10 min or 50 min at 37°C in HeLa cells treated siRNAs against SNX12. Cells were labeled with anti-EEA1 or Lamp1 antibodies and analyzed by triple channel fluorescence. (**E**) HeLa cells were treated with siRNAs against SNX12 or mock treated and then incubated with EGF for the indicated time periods. Cell lysates (100 µg) were analyzed by SDS gel electrophoresis and western blotting with antibodies against EGFR or α-tubulin (a-tub)**.**

During vesicular stomatitis virus (VSV) infection, the envelope of endocytosed virions undergoes low pH-mediated fusion with endosomal membranes. We had observed that VSV fusion occurs primarily with internal vesicles of ECV/MVBs, thereby releasing the viral nucleocapsids into the lumen of internal vesicles [28], [29]. These internal vesicles are then delivered to late endosomes, where their back-fusion with the limiting membrane releases the viral RNA into the cytosol, allowing synthesis of viral proteins to proceed. Typically, viral infection is sensitive to conditions that affect transport to late endosomes or late endosome dynamics, including microtubule depolymerization [28], [29] (Figure 3B-C). By contrast, in SNX3-depleted cells, microtubule depolymerization no longer inhibits viral infection, presumably because the viral RNA is released from an earlier endosome in the pathway [25]. However, after SNX12 depletion (Figure 3A-C), VSV infection remained sensitive to microtubule depolymerization much like in mock-treated cells (Figure 3C, quantification in 3B).

These observations may indicate that SNX12 and SNX3 exhibit different functions. Alternatively, SNX12 and SNX3 may exhibit similar functions, but SNX12 depletion may have little impact given the low levels of its transcript when compared to SNX3 (Figure 1B). Similarly, SNX12 depletion did not affect the transport of EGFR from early-to-late endosomes (Figure 3D) and the degradation of EGFR after continuous EGF stimulation (Figure 3E).

### Ultrastructure of Early Endosomes

**Figure 4:**
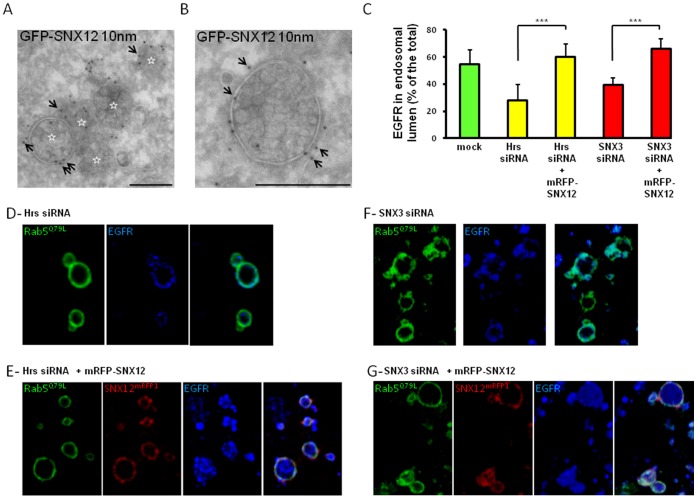
SNX12 overexpression induces an accumulation of MVB-like structures and rescues the intralumenal vesicles that incorporate the EGF receptor. (**A-B**) HeLa cells expressing GFP-SNX12 were fixed and processed for electron microscopy. Cryosections were labeled with antibodies against GFP followed by protein A-gold 10 nm (arrows). Panel A shows a cluster of several multivesicular endosomes, each being labeled with a star, and panel B shows a high magnification view of an individual multivesicular endosome. Scale bar indicates 250 nm. (**C**) Hrs (**D-E**) or SNX3 (**F-G**) was knocked down and mRFP-SNX12 (red) was overexpressed (**E-G**) or not (**D-F**). In each condition, HeLa cells were also transfected with GFP-Rab5^Q79L^ (green) during the last 24 h. After cell surface binding, EGF was internalized for 15 min at 37°C. Cells were processed for immunofluorescence with anti-EGFR antibodies (blue). The relative amount of EGFR in the lumen of endosome was quantified as previously described [25]. Results are expressed as the percentage of the total amount of EGFR. Each condition is the mean of at least three independent experiments; standard errors are indicated and results were analyzed by paired t test (***, p<0,001). (**D-G**) Representative pictures of experiments quantified in (**C**). GFP-Rab5^Q79L^ enlarged endosomes in each condition showed the EGFR localization after Hrs (**D-E**) or SNX3 (**F-G**) was knocked down and mRFP-SNX12 (red) was overexpressed (**E-G**) or not (**D-F**).

Since SNX12 overexpression inhibited transport beyond early endosomes towards late endosomes and lysosomes, we analyzed whether the ultrastructure of the early endosomes was affected. By electron microscopy, clusters containing several multivesicular endosomes were typically observed in cells overexpressing SNX12 (Figure 4A), like SNX3 [25], while such clusters were not observed in control cells (not shown) [3]. Each individual multivesicular element, however, resembled ECV/MVBs, which function as transport intermediates between early and late endosomes. Immunogold labeling showed that the limiting membrane of these structures was decorated with GFP-SNX12, particularly in electron-dense flat regions of the limiting membrane (Figure 4B) resembling the endosomal clathrin coat involved in sorting ubiquitinated proteins into intralumenal vesicles of early endosomes [30], [31]. Since SNX12 is primarily located on early endosomes (Figure 1), these ECV-MVB-like structures presumably correspond to the vesicular regions of early endosomes that cannot detach or mature from early endosomes anymore, perhaps because SNX12 accumulation interferes with coat disassembly.

Altogether, these results suggest that overexpressed SNX12, much like overexpressed SNX3 [25], prevents the detachment of ECV/MVBs, without interfering with the formation of intralumenal vesicles, thereby inhibiting early-to-late endosome transport.

### Sorting of EGFR into the Multivesicular Endosome

In our previous studies, we had found that SNX3 depletion interferes with viral infection because the formation of intralumenal vesicles in ECV/MVBs is inhibited [25]. In these cells, VSV undergoes low pH-fusion with the limiting membrane, since ECV/MVBs are devoid of intralumenal vesicles and thus no longer multivesicular, bypassing the need for microtubule-dependent transport towards late endosomes. Consistent with this view, we had also found that SNX3 depletion inhibits EGFR sorting into empty “multivesicular” endosomes [25], as expected. It had been shown that Hrs depletion also inhibits intralumenal vesicle formation, presumably because Hrs is necessary to recruit ESCRTs and other factors [31], [32], [33]. We found that, in an Hrs knockdown background, SNX3 restores both intralumenal vesicle formation and EGFR sorting into multivesicular endosomes [25].

To determine whether SNX12 could fulfill the same role as SNX3, we investigated whether SNX12 could also restore EGFR sorting into multivescular endosomes after Hrs knockdown. To this end, we used the Rab5^Q79L^ GTPase-deficient mutant of Rab5 to trigger the formation of enlarged endosomes [25], [31], [34] with sufficient spatial resolution for light microscopy analysis (Figure 4D-G, quantification in Figure 4C). As expected, EGFR remained on the limiting membrane of these enlarged endosomes after Hrs or SNX3 depletion (Figure 4D-F, quantification in Figure 4C), presumably because they lacked intralumenal membranes. By contrast, overexpression of SNX12 in the Hrs or SNX3 knockdown background restored the appearance of EGFR in the lumen of enlarged endosomes (Figure 4E-G, quantification in Figure 4C), as did SNX3 [25]. Altogether these results indicate that SNX12 can fulfill the same functions as SNX3 in endosomal membrane transport.

## Materials and Methods

### Cells, Antibodies and Reagents

The maintenance of HeLa, HepG2, Caco-2 and PC-3 cells was as described [3], [35], [36]. We used the following mouse monoclonal antibodies against: GFP (Roche Diagnostics); transferrin receptor (Zymed Laboratories. South San Francisco, CA); EEA1 (Transduction Laboratories); LAMP1 (CD107a; BD Biosciences); EGFR (BD Biosciences); α-tubulin (Sigma- Aldrich). Mouse antibodies against anti-LBPA [37] and anti-VSV-G (17.2.21.4) were described [38]. We also used polyclonal antibodies made in rabbit against Rab6 (Santa Cruz Biotechnology) and made in sheep against the EGFR (Fitzgerald). We are very grateful to Wanjin Hong (Singapore. Singapore) for rabbit polyclonal antibody against SNX3. HRP-labeled secondary antibodies were from Amersham [39] or Sigma-Aldrich (St Louis, MO) and fluorescently labeled secondary antibodies from Jackson Immunoresearch Laboratories (West Grove, PA). Wortmannin, nocodazole, EGF were from Sigma-Aldrich, EGF-biotin-streptavidin-Alexa Fluor® 488 complex and transferrin-Alexa Fluor® 546 from Invitrogen. We are very grateful to Ludger Johannes (Paris. France) for supplying us with Cy3-labeled Shiga toxin B-subunit. We obtained pGFP-Rab5^Q79L^ from Marino Zerial (Dresden. Germany) and myc-tagged SNX3 from Carol R. Haft (National Institutes of Health, Bethesda, Maryland).

### Plasmids, RNA Interference and Transfection

Total RNA from HeLa cells was extracted with phenol-chloroform and precipitated with isopropanol. Then, reverse transcription was performed from 0.5 µg total RNA using Superscript RT (Invitrogen) and random hexamers. Next, the sequence encoding human SNX12 was amplified by PCR using the following primers: 5′-CCGGAATTCCGGATGTCGGACACGGCAGTAGCTG-3′ and 5′-CGGGATCCGCTACTGGCGCACCTTCCCCGGGA-3′ and introduced at EcoRI/BamHI restriction sites into pEGFP-C2 vector (Clontech). SNX12 was also fused with mRFP1 (pmRFP1) or double myc tag (pDMyc). The PtdIns3P binding-defective mutant SNX12^R71A^ was obtained by QuikChange Site-Directed Mutagenesis (Stratagene). The resulting plasmids were verified by DNA sequencing. Cells were transfected with these different vectors using FUGENE® 6 Transfection Reagent (Roche Diagnostics Basel, Switzerland) according to the manufacturer’s instructions. In RNA interference (RNAi) experiments, cells were transfected twice at a 24 h interval using LipofectamineTM RNAiMAX transfection Reagent (Invitrogen) with 21-nucleotide RNA duplexes (Qiagen), replated 4 h after the second transfection, and analyzed 36 h later. For SNX12 silencing, we used the following target SNX12 sequence: 5′-AAGGGATCTTTGAGGAGTCTT-3′.

### Quantitative PCR

Total RNA from different cell types was extracted with RNeasy (Qiagen), according to manufacturer’s instructions. Then, reverse transcription was performed from 5 µg total RNA using Superscript RT (Invitrogen) and Oligod(T) (Invitrogen). SNX3 and SNX12 were amplified by quantitative real time-PCR using SYBR Green (Roche). All real-time PCR reactions were performed on a LC480 (Roche), with the following cycling parameters: annealing and amplification at 60°C for 60 s, and dissociation at 95°C for 10 s. The PCR program was followed by a melting curve and SNX12 values were normalized to the relative amounts of SNX3.

### In vivo Endocytic Transport Assay

HeLa cells were incubated for 1 h at 4°C with 0.5 µg/mL EGF-biotin-streptavidin-Alexa Fluor®^488^ complex, 1 µM Cy3-labeled Shiga toxin B-subunit, or with 20 µg/mL transferrin-Alexa Fluor®^546^. After washes, the marker was then endocytosed for the indicated time periods at 37°C and cells were processed for immunofluorescence. For EGFR degradation, cells were preincubated in serum-free medium, and then incubated with 0.25 µg/mL EGF and 10 µg/mL cycloheximide for the indicated time periods. Cell lysates were analyzed by SDS gel electrophoresis and western blotting with the indicated antibodies.

### Immunofluorescence

Immunofluorescence microscopy has been previously described [40]. Briefly, cells were fixed in 4% PFA for 20 min at room temperature. They were then incubated with the primary antibody in PBS containing 10% FCS and 0.05% saponin for 60 min followed by the Cy2-, Cy3- or Cy5-conjugated fluorescent secondary antibodies. Pictures were captured using a Zeiss LSM 510 META confocal microscope equipped with a 633 Plan-Apochromat objective.

### VSV Infection

The VSV infection was measured by monitoring the appearance of the viral glycoprotein G in the host-cell biosynthetic pathway as described [29]. Briefly, virus (1 MOI) was bound to the plasma membrane on ice and then cells were incubated for 3 h at 37°C. Finally, G-protein appearance in biosynthetic membranes was visualized by immunofluorescence using anti-VSV-G antibodies.

### Liposome Binding Assay

Lipids in chloroform were mixed in a pyrex test tube at the given molar ratios and dried under Nitrogen. Liposome buffer (50 mM Hepes-NaOH pH 7.2, 100 mM NaCl) was added to the dried lipid mixture to a final 0.5 µg/µL lipid concentration, and lipids were rehydrated for 1 h on ice in the dark. After sonication in a bath-type sonicator for 2× 5 min, the lipids were vortexed for 30 s. 50 µg liposomes were mixed with 10 µg protein in 200 µL final volume and incubated for 2 h at 4°C with end-over-end mixing in the dark. Then, the mixture was adjusted to 40.6% sucrose, loaded at the bottom of a TLS-55 centrifuge tube, overlaid sequentially with 35 and 8.5% sucrose solutions in 3 mM imidazole, pH 7.4, and centrifuged in a TLS-55 rotor at 55000 rpm for 1 h at 4°C [41]. The 8.5/35% interphase that contains liposomes, the 35% fraction, as well as the 40.5% fraction that contains the free proteins were collected. Half of the collected samples was analyzed by immunoblotting.

### GST Pull-down

Culture dishes (10 cm) with confluent HeLa cells were washed twice with ice-cold PBS on ice and scraped in lysis buffer containing 20 mM Tris pH 7.4, 150 mM NaCl, 1 mM EDTA, 10% glycerol, 1% digitonin (w/v) and protease inhibitors. After 30 min incubation at 4°C, cell lysates were cleared by centrifugation at 13,000 g for 20 min at 4°C, and then incubated with 25 µg GSTtagged protein overnight at 4°C with end-over-end mixing. The mixture was added to 100 µL glutathione Sepharose beads and incubated 1 h at 4°C with end-over-end mixing. Beads were spun down at 3000 rpm, 3 min at 4°C and then washed 6× in detergent-free lysis buffer. Proteins were eluted from the beads by boiling in sample buffer with β-mercaptoethanol and followed by SDS-gel electrophoresis and western blotting.

## Supporting Information

Figure S1 **EGFR and Shiga toxin B-subunit transport in control cells.** (**A-C**) After cell surface binding, EGF-biotin coupled to streptavidin^AlexaFluor488^ was endocytosed for 10 min (**A**) or 50 min (**B-C**) at 37°C, as in Fig1 but in untransfected HeLa cells. Cells were labeled with anti-EEA1 (**A-B**) or Lamp1 (**C**) antibodies and analyzed by immunofluorescence. (**D**) After cell surface binding, Shiga toxin B-subunit conjugated to Cy3 was internalized for 50 min at 37°C, as in Fig1 but in untransfected HeLa cells. Cells were labeled with Rab6 antibodies and analyzed by immunofluorescence. Bar: 10 µm.
(PDF)

Figure S2 **SNX12 distribution depends on the binding to PtdIns3P.** (**A**) HeLa cells expressing GFP-SNX12 or the 3-phosphoinositide binding defective mutant GFP-SNX12^R71A^ were treated or not with 100 nM wortmannin for 30 min at 37°C and then analyzed by immunofluorescence microscopy. Bar: 10 µm. (**B**) Purified recombinant wild type SNX12 (SNX12 WT) and SNX12^R71A^ mutant were analyzed by SDS gel electrophoresis and visualized after Coomassie blue staining. (**C**) Wild type SNX12 or SNX12^R71A^ were incubated with liposomes containing PtdIns3P or PtdIns. Liposomes were then separated from unbound protein by floatation in a step sucrose gradient. 110 µL from each fraction were collected and 50 µL of them were run on 12% SDS gel and further analyzed by immunoblotting. (**D**) HeLa cells were transfected with GFP-SNX12^R71A^ mutant or mock-treated and then incubated with EGF for the indicated time periods. Cell lysates (100 µg) were analyzed by SDS gel electrophoresis and western blotting with antibodies against EGFR or GFP**.**
(PDF)

Figure S3 **SNX12 interacts with the retromer.** (**A**) HeLa cells expressing mRFP1-SNX12 were processed for immunofluorescence using the indicated antibodies. Scale bar indicates 10 µm. (**B**) Recombinant GST-SNX1, GST-SNX3 and GST-SNX12 were produced, analyzed by SDS gel electrophoresis and visualized after Coomassie blue staining. (**C**) Recombinant proteins visualized in (**B**) were incubated with cell lysates (input), then retrieved using glutathione Sepharose beads and analyzed by SDS gel electrophoresis and western blotting with antibodies against Vps35. Ponceau staining showed the recombinant proteins precipitated after pull down.
(PDF)
