# Supplementary figures and images for: SNX12 Role in Endosome Membrane Transport
Source: PLoS One. 2012 Jun 15;7(6):e38949. doi: 10.1371/journal.pone.0038949 (PMC3376135; doi:10.1371/journal.pone.0038949)

Figure-S1 (Pons)

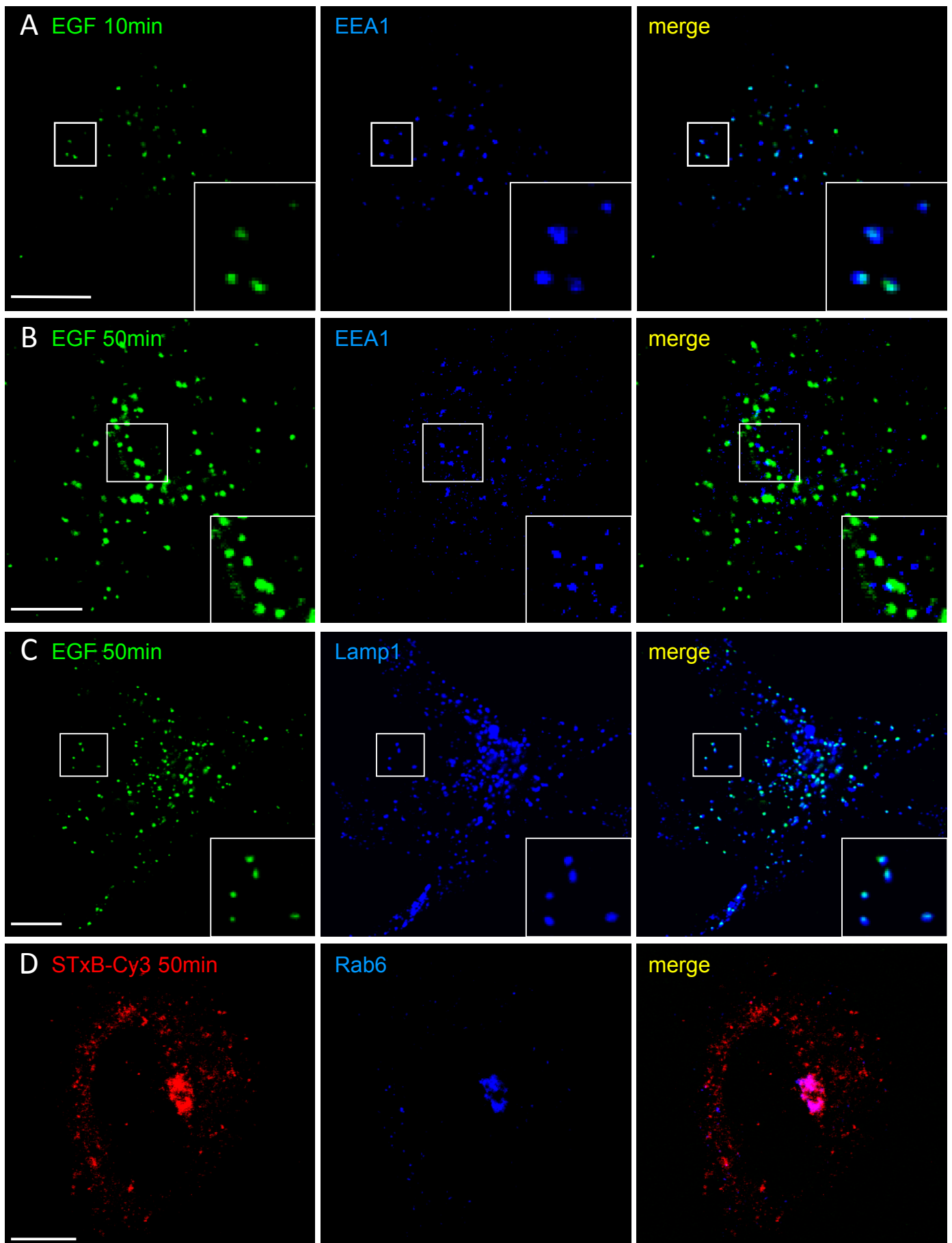

Supplement: Figure S1 — EGFR and Shiga toxin B-subunit transport in control cells. (A-C) After cell surface binding, EGF-biotin coupled to streptavidinAlexaFluor488 was endocytosed for 10 min (A) or 50 min (B-C) at 37°C, as in Fig1 but in untransfected HeLa cells. Cells were labeled with anti-EEA1 (A-B) or Lamp1 (C) antibodies and analyzed by immunofluorescence. (D) After cell surface binding, Shiga toxin B-subunit conjugated to Cy3 was internalized for 50 min at 37°C, as in Fig1 but in untransfected HeLa cells. Cells were labeled with Rab6 antibodies and analyzed by immunofluorescence. Bar: 10 µm. (PDF) [file pone.0038949.s001.pdf]

Figure-S2 (Pons)

A

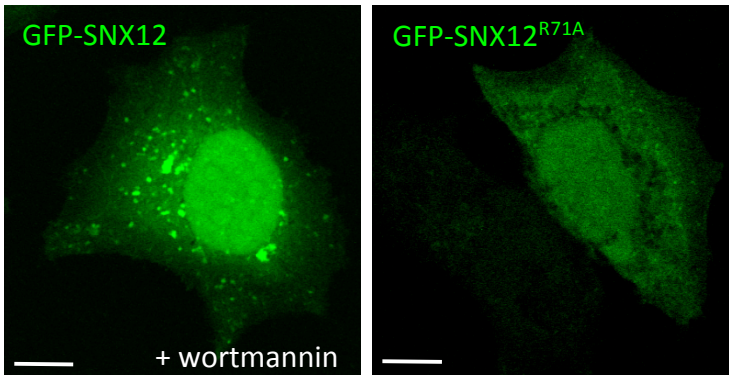

B

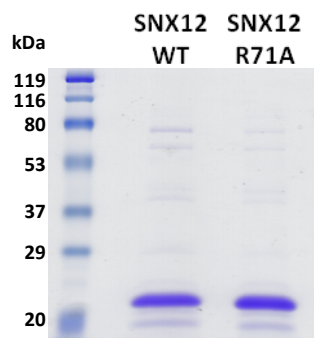

C

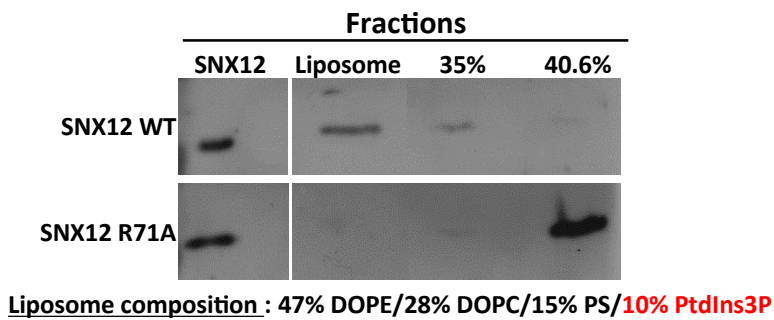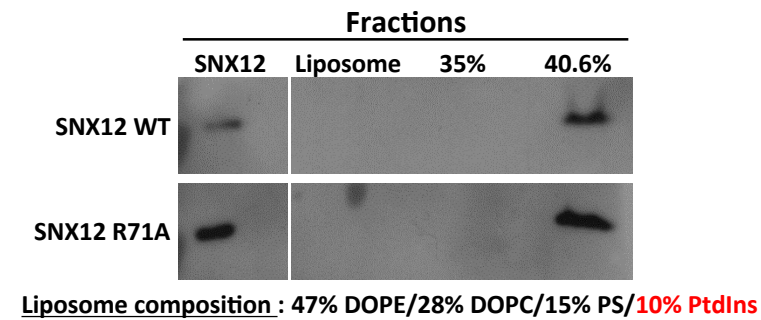

D

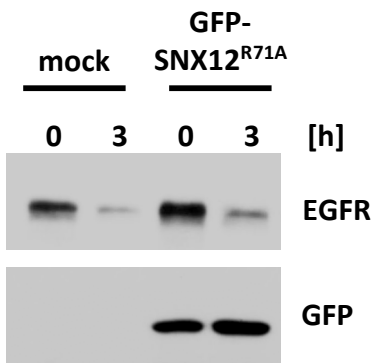

Supplement: Figure S2 — SNX12 distribution depends on the binding to PtdIns3P. (A) HeLa cells expressing GFP-SNX12 or the 3-phosphoinositide binding defective mutant GFP-SNX12R71A were treated or not with 100 nM wortmannin for 30 min at 37°C and then analyzed by immunofluorescence microscopy. Bar: 10 µm. (B) Purified recombinant wild type SNX12 (SNX12 WT) and SNX12R71A mutant were analyzed by SDS gel electrophoresis and visualized after Coomassie blue staining. (C) Wild type SNX12 or SNX12R71A were incubated with liposomes containing PtdIns3P or PtdIns. Liposomes were then separated from unbound protein by floatation in a step sucrose gradient. 110 µL from each fraction were collected and 50 µL of them were run on 12% SDS gel and further analyzed by immunoblotting. (D) HeLa cells were transfected with GFP-SNX12R71A mutant or mock-treated and then incubated with EGF for the indicated time periods. Cell lysates (100 µg) were analyzed by SDS gel electrophoresis and western blotting with antibodies against EGFR or GFP. (PDF) [file pone.0038949.s002.pdf]

Figure-S3 (Pons)

A

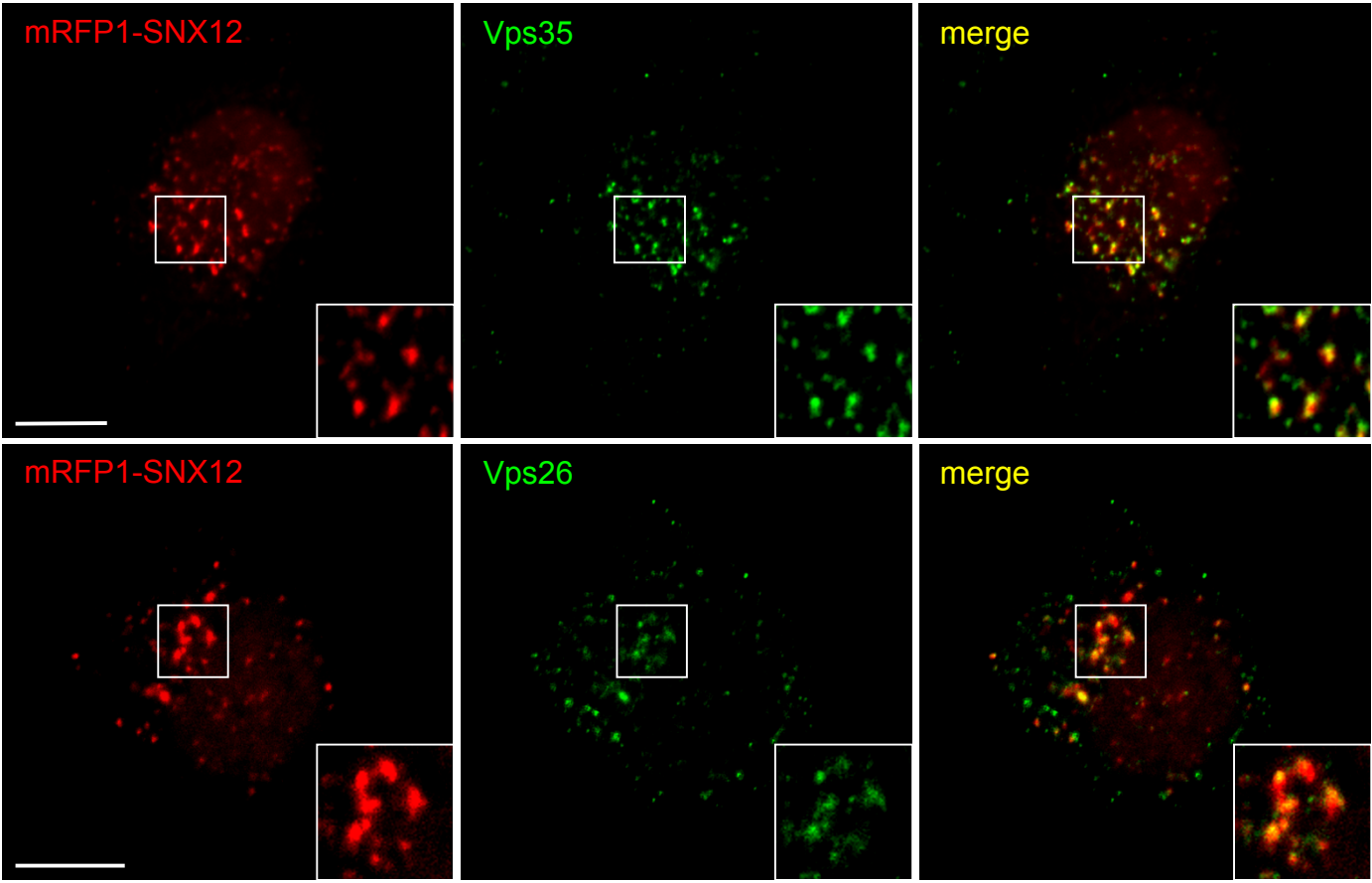

B

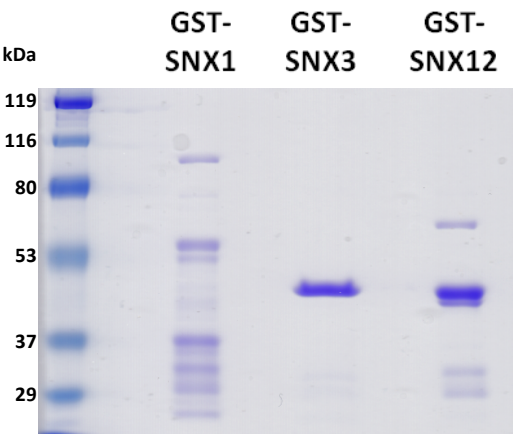

C

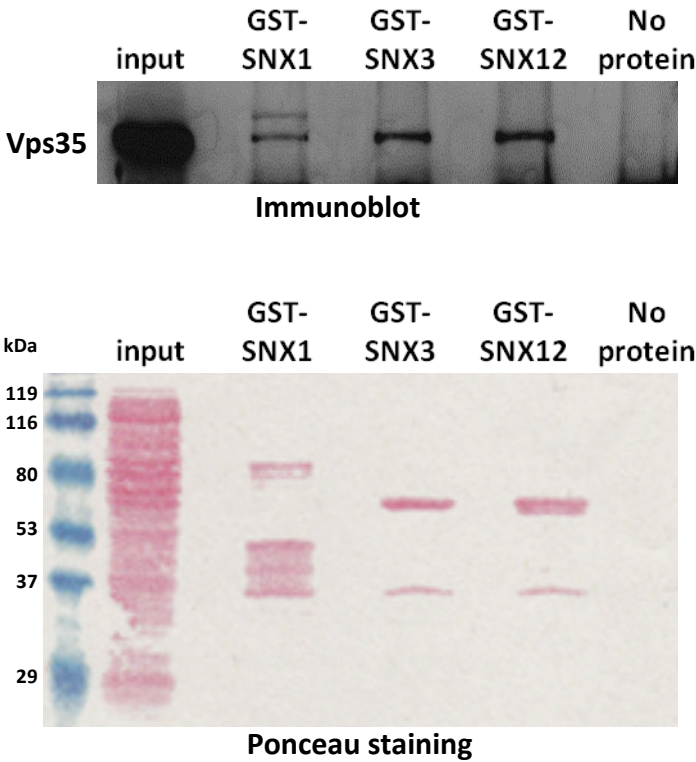

Supplement: Figure S3 — SNX12 interacts with the retromer. (A) HeLa cells expressing mRFP1-SNX12 were processed for immunofluorescence using the indicated antibodies. Scale bar indicates 10 µm. (B) Recombinant GST-SNX1, GST-SNX3 and GST-SNX12 were produced, analyzed by SDS gel electrophoresis and visualized after Coomassie blue staining. (C) Recombinant proteins visualized in (B) were incubated with cell lysates (input), then retrieved using glutathione Sepharose beads and analyzed by SDS gel electrophoresis and western blotting with antibodies against Vps35. Ponceau staining showed the recombinant proteins precipitated after pull down. (PDF) [file pone.0038949.s003.pdf]
